# Serial thrombin generation and exploration of alternative anticoagulants in critically ill COVID-19 patients: Observations from Maastricht Intensive Care COVID Cohort

**DOI:** 10.3389/fcvm.2022.929284

**Published:** 2022-10-06

**Authors:** Tom W. van de Berg, Mark M. G. Mulder, Teba Alnima, Magdolna Nagy, Rene van Oerle, Erik A. M. Beckers, Tilman M. Hackeng, Anne-Marije Hulshof, Jan-Willem E. M. Sels, Yvonne M. C. Henskens, Iwan C. C. van der Horst, Hugo ten Cate, Henri M. H. Spronk, Bas C. T. van Bussel, MaastrICCht Collaborators

**Affiliations:** ^1^Department of Biochemistry, Cardiovascular Research Institute Maastricht, Maastricht University, Maastricht, Netherlands; ^2^Department of Internal Medicine, Maastricht University Medical Centre+, Maastricht, Netherlands; ^3^Department of Intensive Care Medicine, Maastricht University Medical Centre+, Maastricht, Netherlands; ^4^Department of Internal Medicine, Radboud University Medical Centre, Nijmegen, Netherlands; ^5^Cardiovascular Research Institute Maastricht (CARIM), Maastricht University, Maastricht, Netherlands; ^6^Central Diagnostic Laboratory, Maastricht University Medical Centre+, Maastricht, Netherlands; ^7^Department of Cardiology, Maastricht University Medical Centre+, Maastricht, Netherlands; ^8^Thrombosis Expertise Centre Maastricht, Maastricht University Medical Centre+, Maastricht, Netherlands; ^9^Care and Public Health Research Institute, Maastricht University, Maastricht, Netherlands

**Keywords:** thrombin generation, COVID, anticoagulation, ICU, DOACs

## Abstract

**Background:**

COVID-19 associated coagulopathy (CAC) is associated with an increase in thromboembolic events. Current guidelines recommend prophylactic heparins in the management of CAC. However, the efficacy of this strategy in the intensive care population remains uncertain.

**Objective:**

We aimed to measure thrombin generation (TG) to assess CAC in intensive care unit (ICU) patients receiving thromboprophylaxis with low molecular weight heparin (LMWH) or unfractionated heparin (UFH). In addition, we performed statistical modeling to link TG parameters to patient characteristics and clinical parameters. Lastly, we studied the potency of different anticoagulants as an alternative to LMWH treatment in *ex vivo* COVID-19 plasma.

**Patients/Methods:**

We included 33 patients with confirmed COVID-19 admitted at the ICU. TG was measured at least twice over the course of 6 weeks after admission. Thrombin generation parameters peak height and endogenous thrombin potential (ETP) were compared to healthy controls. Results were subsequently correlated with a patient characteristics and laboratory measurements. *In vitro* spiking in TG with rivaroxaban, dabigatran, argatroban and orgaran was performed and compared to LMWH.

**Results:**

Anti-Xa levels of all patients remained within the therapeutic range throughout follow-up. At baseline, the mean (SE) endogenous thrombin potential (ETP) was 1,727 (170) nM min and 1,620 (460) nM min for ellagic acid (EA) and tissue factor (TF), respectively. In line with this we found a mean (SE) peak height of 353 (45) nM and 264 (96) nM for EA and TF. Although fluctuating across the weeks of follow-up, TG parameters remained elevated despite thromboprophylaxis. *In vitro* comparison of LMWHs and direct thrombin inhibitors (e.g., agratroban, dabigatran) revealed a higher efficacy in reducing coagulation potential for direct thrombin inhibition in both ellagic acid (EA) and tissue factor (TF) triggered TG.

**Conclusion:**

In a sub-group of mechanically ventilated, critically ill COVID-19 patients, despite apparent adequate anti-coagulation doses evaluated by anti-Xa levels, thrombin generation potential remained high during ICU admission independent of age, sex, body mass index, APACHE II score, cardiovascular disease, and smoking status. These observations could, only partially, be explained by (anti)coagulation and thrombosis, inflammation, and multi-organ failure. Our *in vitro* data suggested that direct thrombin inhibition compared with LMWH might offer an alternate, more effective anticoagulant strategy in COVID-19.

## Introduction

COVID-19 associated coagulopathy (CAC) has become increasingly evident with high rates of venous thromboembolism (VTE), particularly in severely affected patients (1–3). Therefore, national and international clinical guidelines advocated increased doses of low molecular weight heparin (LMWH) thromboprophylaxis in high-risk patients (4, 5). However, whether this strategy is effective enough in attenuating the hypercoagulable state and thus reducing risk of thrombosis remains unclear (6).

Both the clinical phenotype and the biochemical characteristics of CAC are markedly different from other coagulopathies (7–11). SARS-CoV-2 specific activated coagulopathy-related pathways interacting with host inflammation appear to be important drivers for CAC (9, 12). In addition to hypercoagulability, hypofibrinolysis also plays a role (7, 13–17), possibly explaining mixed results reported for associations between fibrinogen and d-dimer concentrations with thromboembolism (18–20). Furthermore, increased heparin resistance has been observed in severe COVID-19 disease (21–25).

Therefore, more insight into the underlying mechanisms of CAC by which heparins do (or do not) protect against thromboembolism is required. More insight will indicate alternative ways to improve thromboprophylaxis and clinical outcome in SARS-CoV-2 infected patients who experience a severe disease course.

In COVID-19 disease, increased values in the plasma thrombin generation have been associated with a worse prognosis (26, 27). Thrombin generation is a suitable tool to monitor coagulation potential in SARS-CoV-2 infection and has been assessed and adapted for CAC (28). Several reports suggest that thrombin generation is enhanced in CAC (17, 29, 30), independent of heparin treatment (17). However, other studies showed no differences in thrombin generation during various stages of disease severity in SARS-CoV-2 infection, suggesting coagulation exhaustion (31–33). In this paper the results of serial thrombin generation experiments, performed in a cohort of severely COVID-19 patients, admitted to the intensive care unit (ICU) for mechanical ventilation, are presented.

We hypothesized that thrombin generation is enhanced in subjects with CAC, despite treatment with unfractionated heparin (UFH) and LMWH (either prophylactic or therapeutic) and that alternative anticoagulants directed against thrombin or factor Xa might be more beneficial in attenuation of the hypercoagulable state in severe COVID-19 infection.

## Methods

The manuscript was written following the STrengthening the Reporting of Observational studies in Epidemiology (STROBE) guidelines (34).

### Study population

The Maastricht Intensive Care COVID (*MaastrICCht*) study is a prospective cohort study on patients with confirmed COVID-19 admitted to the ICU of the Maastricht University Medical Centre (MUMC+). The design has been described extensively elsewhere (35) and includes comprehensive serial hemostasis and coagulation phenotyping (13). The local institutional review board [Medisch Ethische Toetsingscomissie (METC) 2020-1565/300523] of the MUMC+ approved the study, which was performed based on the regulations of Helsinki. The study is registered in the Netherlands Trial Register (registration number NL8613).

This study included all participants with respiratory insufficiency requiring mechanical ventilation and at least one real-time polymerase chain reaction (RT-PCR) positive for SARS-CoV-2 RNA and a chest CT scan strongly suggestive for SARS-CoV-2 infection, based on a CORADS-score of 4–5 scored by a radiologist (36). Participants were followed until the primary outcome was reached (i.e., either died in the ICU or discharged from ICU). Every day, a comprehensive and uniform set of clinical, physiological, and laboratory variables was collected, reducing the chance of missing data. In addition, when patients were not available for blood withdrawal or laboratory testing failed, the measurement would be rescheduled for the next blood withdrawal.

### Clinical, physiological variables

ICU care for COVID-19 was standardized as described extensively elsewhere (35). Medical history for cardiovascular disease (defined as: diabetes mellitus, myocardial infarction, hypertension, peripheral vascular disease) was scored at ICU admission. APACHE-II score on ICU admission and SOFA-score during ICU stay were calculated. Computed tomography pulmonary angiography (CTPA) was performed when pulmonary embolism was clinically suspected. Due to logistical reasons no routine compression ultrasonography for detection of deep venous thrombosis was performed as described (18).

### Anticoagulation

During the early COVID-19 pandemic, incident thrombosis frequently appeared, resulting in new thromboprophylaxis recommendations. From March 25th 2020, at the start of the MaastrICCht cohort, the COVID specific thromboprophylaxis dosage in the ICU was Nadroparin 3,800, 5,700 and 7,600 IU for respectively <70, 70–90 and >90 kg. After the release of a new national guideline document on April 23rd 2020, this dose was increased (Nadroparin 5,700, 7,600 and 11,400 IU, respectively) (37). Patients who required therapeutic anticoagulants prior to hospital admission were started on therapeutic LMWH upon ICU admission. Vitamin K antagonists and direct oral anticoagulants (DOACs) were switched to therapeutic LMWH. Patients on extracorporeal membrane oxygenation (ECMO) or continuous renal replacement therapy (CRRT) received UFH, dosed on aPTT [heparin therapeutic range (HTR) 50–80s] and anti-Xa (HTR 0.3–0.7 IU/ml) guidelines (21).

### Hemostasis sub-group

Ninety-four patients were enrolled from March 25th until June 12th in the MaastrICCht cohort. Enrollment for the hemostasis sub-group started later, on April 23rd 2020. During the first wave of COVID-19, 36 patients were included into the hemostasis sub-group. To align patients in their COVID-19 disease course, measurements from each patient were included from the day of intubation onwards. Timing from intubation allows for a fairer comparison between thrombin generation parameters and the disease course severity, where disease severity is defined as the need for mechanical ventilation on the ICU due to COVID-19. From April 23rd 2020 onwards additional thrombin generation assays were performed routinely twice weekly in all included MaastrICCht cohort patients. Patients who were in the ICU before April 23rd 2020 or who were transported from another hospital after intubation were also included, starting thrombin generation measurements from admission from April 23rd 2020 onwards. This means that some patients could be included in their first till the sixth week after intubation. This design has been described more extensively elsewhere (13).

### Blood withdrawal and preparation

Daily arterial blood samples from all patients were collected from an arterial line in 7.2 mg K_2_ EDTA (4.0 ml), serum and 3.2% (w/v) sodium citrate Vacutainer blood collection tubes (Becton Dickinson, Plymouth, UK). Fibrinogen concentrations were measured within 2 h of blood collection in citrated plasma, using a SysmexCS2100i hemostasis analyzer (Sysmex Corporation, Kobe, Hyogo, Japan). Detectable fibrinogen concentration had a maximum of 9 g/L. Concentrations of C-reactive protein (CRP, third generation, Roche Diagnostics, Basel, Switzerland) were measured on the COBAS^®^8000 by Roche Diagnostics in serum. Additional 3.2% (w/v) sodium citrate blood tubes were collected for thrombin generation measurements, twice a week for each patient. Platelet poor plasma (PPP) was obtained using two subsequent centrifugation steps: initial centrifugation of 2,490g for 5 mins, followed by 10,000g for 10 mins. Anti-Xa activity (biophen Heparin LRT; HYPHEN Biomed, Neuville-Sur-Oise, France) was measured on a Sysmex CS2100i (Sysmex Corporation) in COVID-19 patient citrate plasma diluted 1:2 with reference pooled citrate plasma. Anti-Xa activity was determined using a LMWH calibration line (aXa-LMWH; HYPHEN Biomed). UFH activity was subsequently calculated with a previously determined formula: UFH anti-Xa = 1.55 ^*^ LMWH anti-Xa (21).

In the second part of the study a switch to *in vitro* experiments was made. For this purpose a separate COVID-19 pooled plasma was created from patients included in the overarching MaastrICCHt cohort. The pooled plasma had an average LWMH level of 0.3 U/ml was prepared by combining plasma of 40 patients with severe COVID-19 admitted to the ICU.

### Thrombin generation

Thrombin generation is quantified by a thrombin generation curve over time and can be described by five parameters (38, 39). First, the endogenous thrombin potential is defined as the surface area under the thrombin generation curve indicating the total amount of thrombin generation that has been generated over the course of plasma coagulation. Second, the peak height (PH) of the thrombin generation curve indicates the maximal thrombin concentration reached. Lag time (start of the curve), velocity index (upward slope of the curve) and time to tail (end of the curve) are less used parameters (38, 39). Thrombin generation was performed in platelet-poor citrated plasma using the Calibrated Automated Thrombogram (CAT) method (Thrombinoscope BV, Maastricht, The Netherlands). Thrombin generation was optimized for COVID-19 as described extensively elsewhere (28). Briefly, thrombin generation was triggered by either 10 μg/ml ellagic acid (reflecting the intrinsic route of coagulation activation, similar to aPTT) or high tissue factor (reflecting the extrinsic route of coagulation activation, similar to PT) (PPP Reagent HIGH). Phospholipids were added in a 20/60/20 (PS/PC/PE) ratio to the concentration of 4 μM. Thrombin generation was assessed by adding a low-affinity fluorescent thrombin substrate (Z-Gly-Gly-Arg 7-amino-4-methylcoumarin) and utilizing a 390/460 nm filter. Machine setup and data recording were performed using the thrombinoscope software (Thrombinoscope BV, Maastricht, the Netherlands). *In vitro* spiking of normal pooled plasma and COVID-19 pooled plasma was performed by initial addition of 0.3 IU/ml fraxiparin (Mylan BV, Canonsburg, USA) and followed by further addition of 0.3–1.2 IU/ml fraxiparin (Mylan BV, Canonsburg, USA), 0–4 μg/ml argatroban (Goodlife pharma Nederland, Naarden, The Netherlands), 0–1.6 U/ml orgaran (Mylan BV, Canonsburg, USA), 0–320 ng/ml dabigatran (Boehringer Ingelheim bv, Ingelheim am Rhein, Germany) and 0–320 ng/ml rivaroxaban (Bayer BV, Leverkusen, Germany).

### Statistical analyses

Data were analyzed using SPSS version 25. Data were included starting at the date of intubation until a maximum of 6 weeks of ICU admission. The first thrombin generation assay per week was included in the analyzes for each patient (if available). Admission characteristics were described using mean and standard deviation (SD), median and interquartile range or percentage, as appropriate. First, the mean anti-Xa level per week was investigated to detect the presence of LMWH or UFH. Second, the longitudinal trends in thrombin generation parameters were analyzed using linear mixed models, since these account for the interdependency in serial measurements over time within patients. First, in a crude model (model 1), we analyzed the association between time (week 1 to week 6) and thrombin generation parameters (endogenous thrombin generation potential and peak height). Time was treated as a categorical variable. Second, we adjusted thrombin generation data for age, sex, body mass index, APACHE II, cardiovascular disease, and smoking (model 2). Third, models 2 was additionally adjusted to investigate whether serial anticoagulation measurements (i.e., anti-Xa level and fibrinogen) (18), serial inflammation parameters (i.e., C-reactive protein) (9), serial multi-organ dysfunction parameters (40) or incident pulmonary embolism and deep venous thrombosis events could explain the association between time and thrombin generation parameters.

We report regression coefficients β with 95% confidence intervals (95%CI) and considered a *p*-value <0.05 statistically significant.

## Results

### Patient characteristics

We included 33 out of 36 patients from the COVID-19 hemostasis sub-group; from 3 patients no data on thrombin generation were collected. The mean ± SD age was 61.6 ± 9.7 years, 82% were men. The body mass index was 28.0 ± 4.3 kg/m^2^. At ICU admission C-reactive protein was 202 ± 84 mg/L and fibrinogen was 6.6 ± 2.0 g/L. At ICU admission, the APACHE-II score was 16 ± 4, and the SOFA score was 7 ± 1.8. During ICU stay, 19 (58%) patients were diagnosed with pulmonary embolism, 1 patient was diagnosed with deep venous thrombosis (3%). Six patients (18.2%) received ECMO and seven patients (22.2%) received CRRT. The length of stay at the ICU was 33.4 ± 16.2 days, with a mortality rate of 36% (Table 1). Measured samples were included into the study based on weeks after ICU admission, weekly between 10 and 22 individual patient samples were included in the analysis, of which on average 46–92% of patients were treated with therapeutic doses of either LMWH or UFH (Table 2). Anti-Xa levels for the whole group were 0.56 ± 0.23 IU/ml.

**Table 1:** Patient characteristics.

**General**	***n*** **= 33**
Age, year, mean (SD)	61.6 (9.7)
Male, n (%)	27 (81.8)
Body mass index, kg/m^2^, mean (SD)	28.0 (4.3)
**Medical history**	
Diabetes Mellitus type II, n (%)	1 (3%)
Hypertension, n (%)	9 (27.2%)
Malignancy, n (%)	4 (12.1%)
Myocardial infarction, n (%)	1 (3%)
Peripheral vascular disease, n (%)	1 (3%)
Smoker, n (%)	2 (6%)
**Medication prior to admission**	
Immunosuppression, n (%)	0 (0%)
Angiotensin II converting enzyme inhibitors, n (%)	5 (15.2%)
Angiotensin II receptor blocker, n (%)	4 (12.1%)
Calcium channel blockers, n (%)	1 (3%)
β-blockers, n (%)	4 (12.1%)
Diuretics, n (%)	2 (6%)
Lipid lowering agents, n (%)	5 (15.2)
Antiplatelet agents, n (%)	3 (9%)
Coumarins, n (%)	0 (0%)
Direct oral anticoagulants, n (%)	3 (9%)
**ICU events**	
SOFA score at admission, mean (SD)	7.0 (1.8)
APACHE II score at admission, mean (SD)	16.0 (4.4)
Length of ICU stay, days, mean (SD)	33.4 (16.2)
CRRT during ICU stay, n (%)	7 (21.2%)
ECMO during ICU stay, n (%)	6 (18.2%)
**Thrombotic complications at ICU:**	
Deep venous thrombosis, n (%)	1 (3%)
Pulmonary embolism, n (%)	19 (58%)
ICU mortality, n (%)	12 (36%)

SOFA-score, Sepsis-related Organ Failure Assessment; APACHE II score, acute physiology and chronic health evaluation; SAPS II, simplified acute physiology; CRRT, continuous renal replacement therapy; ECMO, extracorporeal membrane oxygenation; ICU, intensive care unit.

**Table 2:** Number of COVID-19 patients, the incidence of therapeutic anticoagulation, routine laboratory measurements, SOFA-score, and anti-Xa levels per week starting from intubation.

	**Week number**					
**Parameters**	**1**	**2**	**3**	**4**	**5**	**6**
Number of patients (n)	13	14	22	16	14	10
Percentage of patients treated with TA (%)	46	68	82	81	92	78
CRP (mg/L)	28 (9–134)	37 (24–115)	83 (41–159)	114 (45–199)	135 (45–231)	305 (220–360)
Fibrinogen (g/L)	5.7 (4.5–7.5)	5.4 (4.8–6.1)	6.1 (5.3–7.9)	7.7 (6.5–8.5)	8.2 (6–9)	8.9 (8.1–9)
SOFA-score	3 (2–5)	6 (4–7)	6 (3–7)	6 (3–8)	6.5 (4–9)	7 (6–8)
Anti-Xa (IU/ml)	0.68 (0.32)	0.57 (0.25)	0.53 (0.11)	0.53 (0.26)	0.58 (0.18)	0.51 (0.2)

TA, therapeutic anticoagulation; CRP, C-reactive protein; SOFA-score, Sepsis-related Organ Failure Assessment.

### Ellagic acid triggered thrombin generation

**Figure 1:**
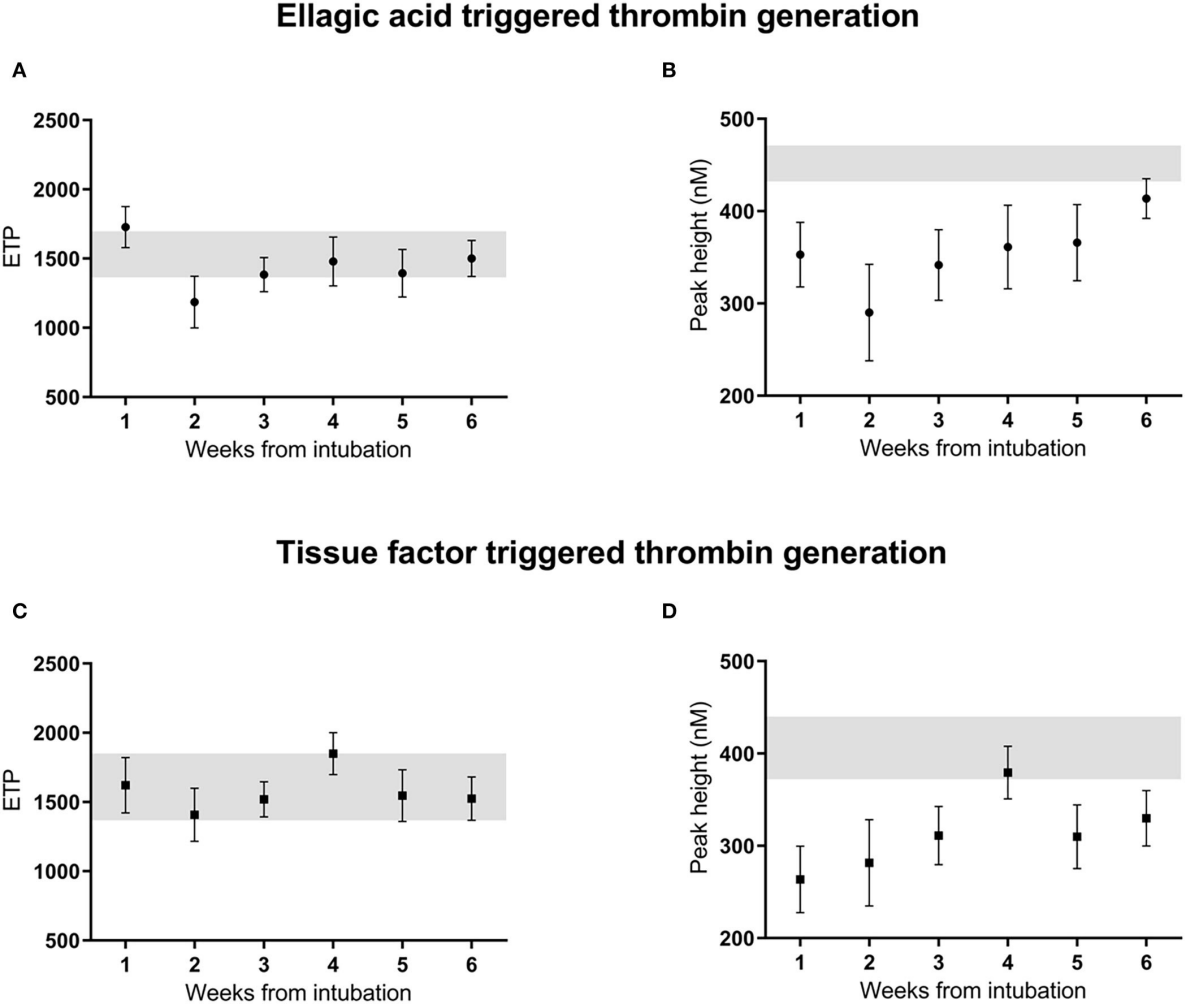
Thrombin generation ETP **(A,C)** and peak height **(B,D)** initiated by ellagic acid and tissue factor, unadjusted measurements over 6 weeks from intubation in critically ill patients with COVID-19 infection. Reference range of normal pooled plasma shown in gray. ETP, endogenous thrombin potential; EA, ellagic acid; TF, tissue factor, A.

The mean (SE) endogenous thrombin potential (ETP) upon ellagic acid activation was 1,727 (170) nM min (Figure 1A) in the 1st week after intubation. Using linear mixed models, ETP in a crude model was significantly lower with −541 nM min (95%CI: −1,011 to −73) in the 2nd week, but in none of the other weeks after intubation compared to the first week (Table 3, model 1). After adjustment for age, sex, body mass index, APACHE-II score, cardiovascular disease, and smoking, ETP was significantly lower by −522 nM min (95%CI: −1,029 to −15) in the 2nd week, but in none of the other weeks after intubation compared to the first week (Table 3, model 2). Additional adjustment of model 2 by serial C-reactive protein, anti-Xa level, fibrinogen, SOFA score, pulmonary embolism and deep venous thrombosis, showed lower ETP in the 2nd [−724 nM min (95%CI: −1,143 to −304), the 3rd [−581 nM min (95%CI: −967 to −196)], and the 5th [−468 nM min (95%CI: −932 to −5)] weeks after intubation compared to the first week (Table 3, model 3). Overall, both statistical models (model 2, model 3) were not associated with significant changes in thrombin generation parameters over the course of the study.

**Table 3:** Ellagic acid-induced thrombin generation parameters, differences over time.

**Model**	**Week 1**	**Week 2**	**Week 3**	**Week 4**	**Week 5**	**Week 6**
**ETP**						
1	Ref	−541 (−1,011 to −71.6)^*^	−342 (−773 to 87.9)	−247 (−703 to 208)	−333 (−802 to 137)	−226 (−739 to 287)
2	Ref	−522 (−1,029 to −14.6)^*^	−179 (−632 to 275)	14.5 (−479 to 508)	−121 (−614 to 371)	191 (−416 to 799)
3	Ref	−724 (−1,143 to −304)^*^	−581 (−967 to −196)^*^	−415 (−840 to 11.2)	−468 (−932 to −5.00)^*^	−270 (−792 to 252)
**Peak height**						
1	Ref	−62.9 (−187 to 61.2)	−11.3 (−125 to 102)	8.23 (−112 to 129)	13.0 (−111 to 137)	60.6 (−74.7 to 196)
2	Ref	−99.2 (−233 to 34.6)	−8.47 (−128 to 111)	39.6 (−90.7 to 170)	24.5 (−105 to 154)	109 (−51.6 to 269)
3	Ref	−158 (−260 to −56.6)^*^	−123 (−216 to −29.1)^*^	−87.0 (−190 to 16.3)	−81.3 (−194 to 31.1)	−25.8 (−152 to 101)

Model 1 = unadjusted. Model 2 = adjusted for age, gender, body mass index, APACHE II, cardiovascular disease, smoking. Model 3 = adjusted according to model 2 + serial C-reactive protein, anti-Xa level, fibrinogen, SOFA score, pulmonary embolisms and deep venous thrombosis.

* P < 0.05 compared to reference.

The mean (SE) peak height (PH) upon by ellagic acid activation was 353 (45) nM (Figure 1B) in the 1st week after intubation. Using linear mixed models, peak height in a crude model showed no statistically significant difference over time since intubation. Adjustment for age, sex, body mass index, APACHE-II score, cardiovascular disease, and smoking status did not change this result (Table 3, models 1–2). Additional adjustment of model 2 by serial anti-Xa level, fibrinogen, C-reactive protein, SOFA score, pulmonary embolism and deep venous thrombosis, showed a reduced peak height in the 2nd [−158 nM (95%CI: −260 to −57)], and in the 3th [−123 nM (95%CI: −216 to −29)] week after intubation compared to the first week (Table 3, model 3).

### Tissue factor triggered thrombin generation

The mean (SE) ETP upon tissue factor trigger was 1,620 (460) nM min (Figure 1C) in the 1st week after intubation. Using linear mixed models ETP in a crude model was not significantly different in weeks 2–6 since intubation compared to the first week. Adjustment for age, sex, body mass index, APACHE-II score, cardiovascular disease, and smoking did not change this result (Table 4, models 1–2). Additional adjustment of model 2 by serial C-reactive protein, anti-Xa level, fibrinogen, SOFA score, pulmonary embolism and deep venous thrombosis, showed a reduced ETP of −470 nM min (95%CI: −890 to −50) in the 3rd week, but in none of the other weeks, after intubation compared to the first week (Table 4, model 3).

**Table 4:** Tissue factor-induced thrombin generation parameters, differences over time.

**Model**	**Week 1**	**Week 2**	**Week 3**	**Week 4**	**Week 5**	**Week 6**
**ETP**						
1	Ref	−212 (−707 to 283)	−102 (−542 to 338)	229 (−245 to 703)	−75 (−549 to 399)	−95 (−604 to 413)
2	Ref	116 (−431 to 663)	106 (−350 to 562)	384 (−105 to 873)	287 (−209 to 782)	500 (−101 to 1,101)
3	Ref	−311 (−790 to 168)	−470 (−890 to −50)^*^	−123 (−570 to 323)	−130 (−629 to 368)	3 (−551 to 557)
**Peak height**						
1	Ref	18.0 (−84.9 to 121)	47.6 (−43.8 to 139)	116 (17.2 to 214)^*^	46.3 (−52.2 to 145)	66.4 (−39.3 to 172)
2	Ref	56.2 (−60.4 to 173)	65.7 (−31.6 to 163)	125 (20.7 to 229)**	93.6 (−12.1 to 199)	138 (10.3 to 267)**
3	Ref	−34.1 (−133 to 64.6)	−65.3 (−152 to 21.3)	14.1 (−77.9 to 106)	−0.95 (−104 to 102)	24.4 (−90.0 to 139)

Model 1 = unadjusted. Model 2 = adjusted for age, gender, body mass index, APACHE II, cardiovascular disease, smoking. Model 3 = adjusted according to model 2 + serial C-reactive protein, anti-Xa level, fibrinogen, SOFA score, pulmonary embolisms and deep venous thrombosis.

* P < 0.05 compared to reference.

The mean (SE) peak height for thrombin generation triggered by tissue factor was 264 (96) nM thrombin (Figure 1D) in the 1st week after intubation. Using linear mixed models the peak height in a crude model, was significantly higher in the 4th week after intubation compared to the reference value [116 nM thrombin (95%CI: 17–214); Table 4, model 1]. After adjustment for age, sex, body mass index, APACHE-II score, cardiovascular disease, and smoking, the peak height was significantly higher in the 4th [125 nM (95%CI: 21–229)] and the 6th week [138 nM (95%CI: 11–267)] after intubation compared to the first week (Table 4, model 2). Additional adjustment of model 2 by serial C-reactive protein, anti-Xa level, fibrinogen, SOFA score, pulmonary embolism and deep venous thrombosis showed no statistically significant difference over the weeks since intubation (Table 4, model 3). Overall, both statistical models (model 2, model 3) were not associated with significant changes in thrombin generation parameters over the course of the study.

Patterns over time for the parameters lag time (start of the curve), velocity index (upward slope of curve) and time to tail (end of curve), were in agreement with results for ETP and peak height for both ellagic acid and tissue factor triggered thrombin generations (data not shown).

### Additional attenuation of coagulation

The obtained thrombin generation data suggest a restricted anticoagulant potential of LMWH in plasma from severe COVID-19 patients. For both the ellagic acid and tissue factor triggered thrombin generation, the ETP for COVID-19 plasma was within the reference range of normal pooled plasma and the peak height was only slightly lower, despite being on therapeutic anticoagulants (Figure 1). Overall, in most assessed COVID-19 plasma aliquots, thrombin generation was only slightly affected by the presence of LMWH.

**Figure 2:**
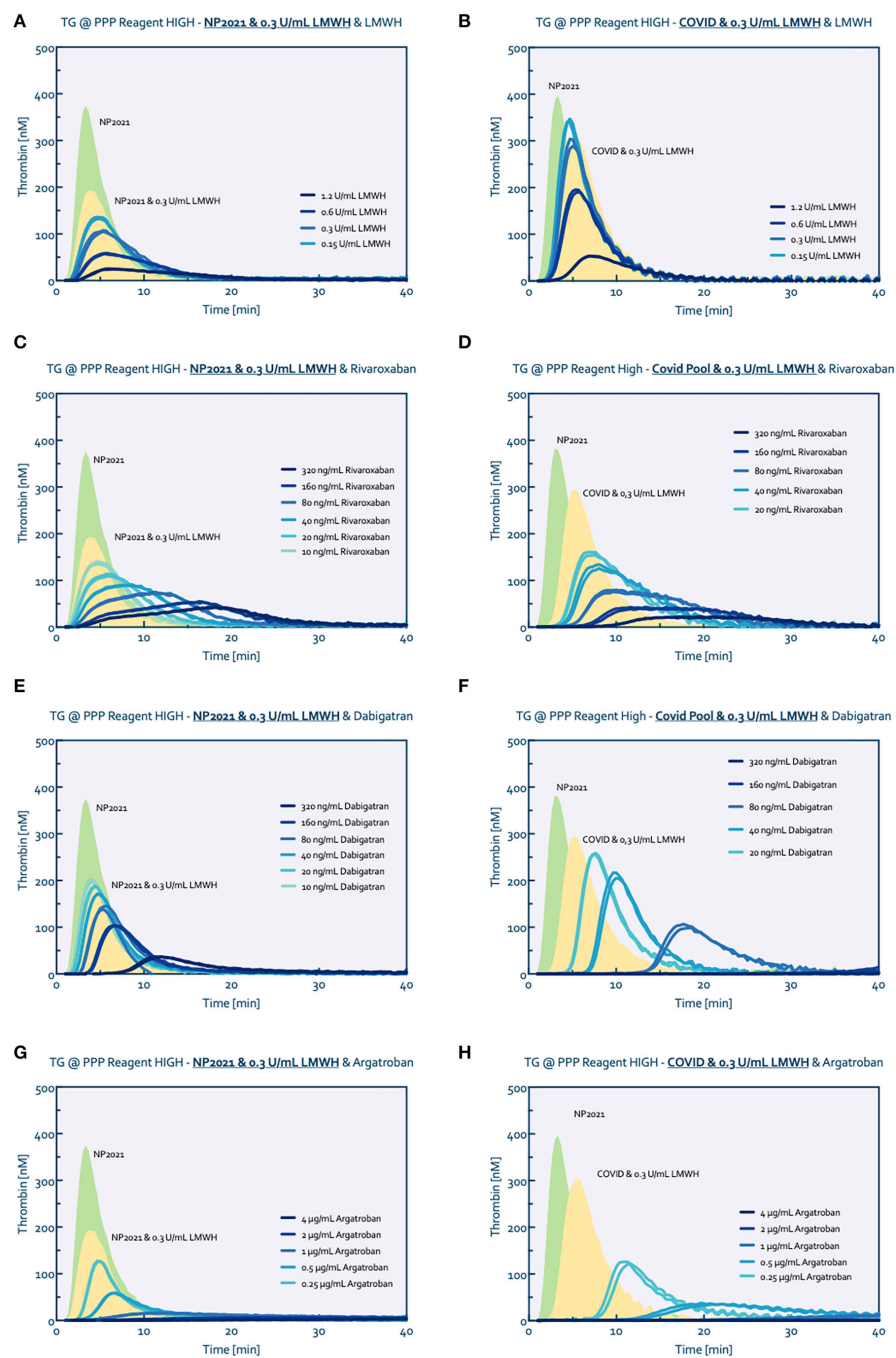
Thrombin generation titrated with several anticoagulants. Normal pool plasma (NP) was measured without (green curve) and with the addition of 0.3 U/mL LMWH (yellow curve) to match the baseline conditions of the COVID pool plasma (0.3 U/ml LMWH). Thrombin generation was consecutively measured after spiking with LWMH **(A,B)**, Rivaroxaban **(C,D)**, Dabigatran **(E,F)** and Argatroban **(G,H)**, respectively.

Comparing normal pooled plasma with COVID-19 pooled plasma (0.3 IU/ml LMWH) both (additionally) spiked with LMWH from 0.15 to 1.2 IU/ml showed an overall higher tissue factor triggered thrombin generation profile for the COVID-19 pooled plasma at all heparin levels (Figures 2A,B). Adding 0.3 IU/ml LWMH to normal pooled plasma reduced the peak height by ~50%. Spiking this plasma with an additional 0.6 IU/ml LMWH reduced the peak height further from 180 to 55 nM thrombin. In comparison, adding 0.6 IU/ml LMWH to COVID-19 pooled plasma (containing 0.3 IU/ml LMWH) reduced the peak height by only ~35% from 300 to 180 nM thrombin.

**Figure 3:**
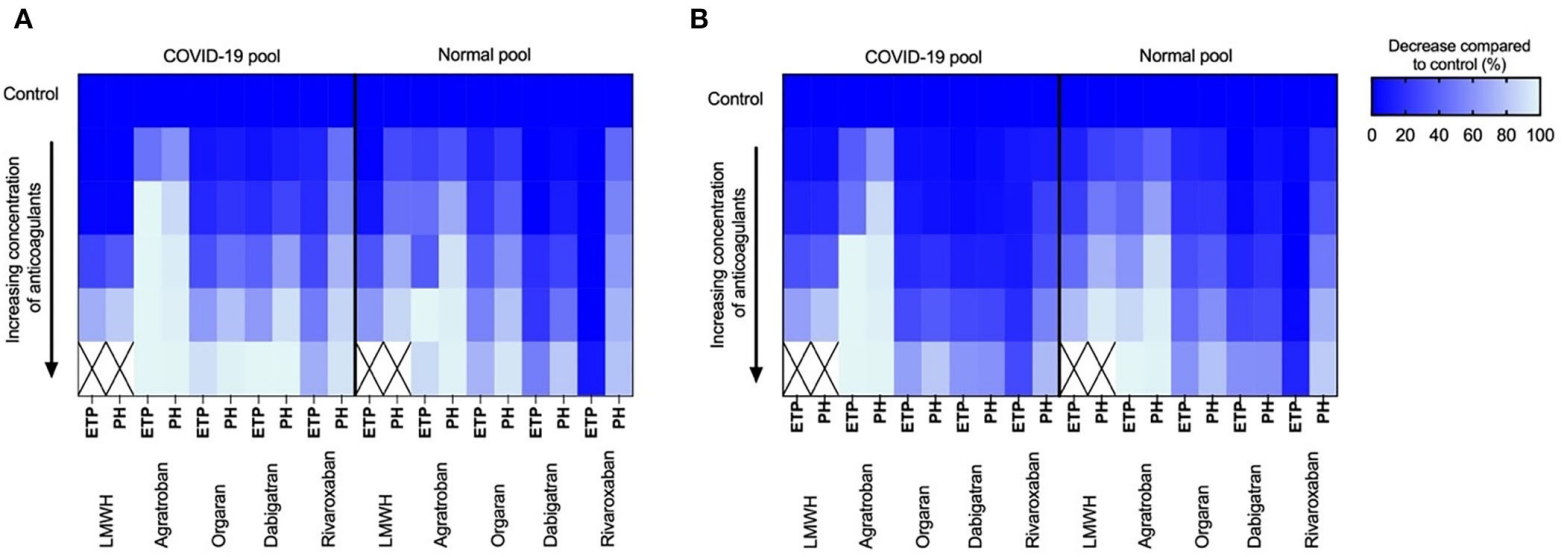
Tissue factor and ellagic acid triggered thrombin generation showing dose dependent inhibition of coagulation by low-molecular-weight-heparins (LMWH; 0.3–1.2 IU/ml), Argatroban (0–4 μg/ml), orgaran (0–1.6 U/ml), dabigatran (0–320 ng/ml) and rivaroxaban (0–320 ng/ml) in normal pool plasma and COVID-19 pool plasma spiked with 0.3 IU/ml LMWH. Color scheme indicates the reduction (0–100%) in endogenous thrombin potential (ETP) and peak height (PH) upon increasing concentration of inhibitors compared to control (100%). **(A)** Inhibition of tissue factor triggered thrombin generation and **(B)** Inhibition of ellagic acid triggered thrombin generation.

Additional inhibition of factor Xa with rivaroxaban on top of the 0.3 IU/ml LMWH showed comparable reduction profiles for both the normal and COVID-19 pooled plasma (Figures 2C,D); similarly, the anticoagulant potential of danaparoid was comparable for normal and COVID-19 pooled plasma (Figure 3).

In contrast, thrombin inhibition by either dabigatran or argatroban in combined with 0.3 IU/ml LMWH showed a higher anticoagulant potential in COVID-19 pooled plasma than the normal pooled plasma (Figures 2E,F for dabigatran, Figures 2G,H for argatroban). The addition of 80 ng/ml dabigatran to COVID-19 pooled plasma prolonged the lag time from 3 to 14 mins and reduced the peak height from 290 nM to 100 nM thrombin. Comparable to dabigatran, 0.25 μg/ml argatroban prolonged the lag time to 8 mins and reduced the peak height to 125 nM thrombin. In comparison, 80 ng/ml dabigatran in normal pooled plasma prolonged the lag time by 1.5–3 mins and reduced the peak height by 50 nM from 190 to 140 nM thrombin. In the presence of 0.25 μg/ml argatroban, thrombin generation in normal pooled plasma (with 0.3 IU/mL LMWH) was only prolonged by 1 minute with a peak height reduced to 130 nM.

Comparable results were obtained for the ellagic acid triggered thrombin generation, with additional anticoagulant potential of thrombin inhibition in COVID-19 pooled plasma (Figure 3).

## Discussion

The study has resulted in five main findings; First, anti-Xa levels remained within therapeutic range throughout the ICU stay despite the varying number of patients on therapeutic and prophylactic anticoagulation per week. Second, the coagulation potential measured by the thrombin generation assay by both ETP and peak height remained high over the course of six weeks after intubation, despite adequate anti-Xa levels. This observation is independent of sex, age, body mass index, APACHE-II, cardiovascular disease, and smoking status. Third, persistently high coagulation potential by thrombin generation was lower after additional adjustment for serial C-reactive protein, anti-Xa level, fibrinogen, SOFA score, incident pulmonary embolism and deep venous thrombosis. This implies, at least a partial role of inflammation, coagulation, and multi-organ failure in the observed association between time and ETP and peak height of thrombin generation in patients on heparin or LMWH. Fourth, the results of persisting high coagulation potential seem to be most evident in ellagic acid-initiated thrombin generation assays. This, in combination with the evidence of contact activation by neutrophil extracellular traps (NETs) in COVID-19 could imply an important role for the intrinsic coagulation cascade and contact activation system in COVID-19 associated coagulopathy. Finally, in vitro addition of thrombin inhibitors to COVID-19 pooled plasma showed a significantly increased anticoagulant potential compared to LMWH only, suggesting a benefit of direct thrombin inhibitors in reducing COVID-19 coagulopathy.

In the present study we have noticed a variable percentage (46–92%) of patients with an indication for therapeutic anticoagulation using LMWH or UFH over the weeks during ICU admission. The accompanying anti-Xa levels were within the therapeutic range suggesting that anticoagulation was effectively dosed. However, whether this dose is effective enough in reducing the incidence of thrombotic complications in COVID-19 patients remains unclear. In the MaastrICCht cohort the incidence of pulmonary thromboembolism is 33% (18), which was in line with other comparable cohorts (20, 41–43) of mechanically ventilated COVID-19 patients. However, our data on thrombin generation suggests that this anticoagulant treatment does not adequately reduce thrombin generation in COVID-19 patients, which may contribute to clinical thrombosis. This is in line with the observations made in other studies (44, 45).

Our analyses revealed that elevated thrombin generation potential persisted over time independent of clinical characteristics and disease severity scores. The ongoing thrombophilic state could only be explained to a small extent by clinical markers of inflammation, coagulation, and multi-organ failure (46). Inflammation, disease severity and thromboembolic complications seem to explain thrombin generation peaks at least partially in the first three weeks of admission as displayed by model 3. In contrast, model 2 showed some changes in weeks 4 and 6 possibly suggesting an influence of patient characteristics in disease recovery.

In addition, the coagulation potential in COVID-19 patients remained high over the course of 6 weeks after admission to the ICU despite adequate anti-Xa levels. However, the coagulation potential of the COVID-19 population would most likely be even higher without LMWH. The inability to normalize the thrombin generation potential may reflect a mechanism leading to heparin resistance, driving the hypercoagulable state in CAC (23, 47). Interestingly, persisting increases in thrombin generation have been reported even up to a year after discharge, possibly caused by underlying mechanisms of persistent endothelial damage (48–51).

We measured thrombin generation initiated by either intrinsic (EA) or extrinsic (TF) activation of coagulation. Although a similar pattern of both ETP and peak height was seen throughout admission, some differences were observed. EA initiated thrombin generation showed significant changes in peak height throughout weeks 2 and 3 whereas the TF initiated thrombin generation did not. Thrombin generation was lower for both ETP and peak height in week 2, which then gradually increased again over time. This pattern over time was similar after adjustment for anti-Xa and C-reactive protein, suggesting an alternative mechanism that drives coagulation potential. Possibly NETs and histones are important as the contact activation pathway is activated by neutrophilic extracellular traps (NETs) and histones. This can lead to increased use of coagulation factors and inhibitors (9). In fact, our models adjusted for clinical markers of inflammation might be too non-specific to detect evidence for such an inflammatory mechanism. Another important observation were the flattened, prolonged thrombin generation curves in combination with a prolonged time to tail. These characteristics can be seen in samples with impaired antithrombin activity. It has been shown that histone citrullination and reduced levels of antithrombin play a significant role in COVID-19 disease compared to non-coronavirus sepsis patients in the ICU (52). However, also antithrombin can be citrullinated, decreasing its effective biological activity even further (53). Lowered and dysfunctional antithrombin could in part explain the insufficient inhibitory potential of heparin in severe COVID-19 patients as heparin inhibits thrombin and FXa by potentiating antithrombin binding. Our findings also support this, showing an insufficient decrease in thrombin generation potential during adequately dosed heparin treatment while we found a potential additional anticoagulant value of anticoagulants acting independently from antithrombin. The hypothesis of increased heparin resistance is further strengthened by recent observations of Benoit et al. who found no significant increases in thrombin generation after neutralization of heparin in COVID-19 patients (54).

Based on our *in-vivo* measurements of thrombin generation we evaluated several other possible anticoagulant treatments *in vitro*. The supplementation with additional anti-thrombin dependent anticoagulants (LMWH, danaparoid) was significantly less effective in COVID-19 patients compared to control plasma, supporting the hypothesis of lowered and dysfunctional antithrombin in these patients. Interestingly direct thrombin inhibitors (dabigatran, argatroban) were more effective anticoagulants in COVID-19 plasma compared to spiked normal pool plasma, possibly due to a high amount of fibrin-bound thrombin which remains biologically active while being shielded from some of the natural inhibitors but not anticoagulants. For this reason, we think that (direct) thrombin inhibitors may offer an attractive therapeutic tool as anticoagulant treatment of CAC and possibly other inflammation-mediated coagulopathies.

To our knowledge, we present one of the few studies describing serial assessment of thrombin generation follow-up for multiple weeks after ICU admission that have been reported in the current literature. Limitations to our study mainly involve its generalizability and applicability in clinical practice. Due to the build-up of our cohort, not all patients were followed from ICU admission. As a result of this, we collected extensive follow-up over a long period, but conclusions concerning individual disease courses cannot be drawn from our data. The inclusion of all mechanically ventilated COVID-19 patients, irrespective of ECMO and CRRT, makes for a heterogeneous population, suggesting careful interpretation of the results and limited generalizability to other ICU populations. Additionally, because of the relatively small number of patients, no conclusions can be drawn regarding the risk for adverse outcome (VTE or mortality), which would have added to the study value. Finally our population is limited to first wave patients, which might hamper translation to patients admitted to ICU in current clinical practice.

## Conclusion

We showed that, in a cohort of mechanically ventilated, critically ill COVID-19 patients on anticoagulation, despite apparent adequate anti-Xa levels, thrombin generation potential remains elevated. Elevated thrombin generation potential persists over the course of ICU admission and is independent of age, sex, body mass index, APACHE II score, cardiovascular disease, and smoking status. These observations may, at least in part, be explained by (anti)coagulation and thrombosis, inflammation, and multi-organ failure states. The lack of anticoagulant efficacy despite adequate anti-Xa levels suggests a mechanism of heparin resistance. Our *in vitro* assays indicate that antithrombin-dependent anticoagulants are relatively ineffective in reducing thrombin generation in COVID-19. In contrast, direct thrombin inhibitors show a promising anticoagulant potential compared to LMWH in severe COVID-19 patients, however more research is needed to confirm this. More in-depth investigation of mechanisms driving thrombin generation potential in COVID-19 is warranted to increase our understanding of CAC.

## Data availability statement

The raw data supporting the conclusions of this article will be made available by the authors, without undue reservation.

## Ethics statement

The studies involving human participants were reviewed and approved by Medisch-Ethische Toetsingscommissie AzM/UM. Written informed consent for participation was not required for this study in accordance with the national legislation and the institutional requirements.

## Author contributions

TvB and MM analyzed and prepared the data and wrote the manuscript. TA performed statistical modeling and wrote a subsection regarding the statistical analysis. MN performed *in vitro* experiments and provided a visual representation of the results. RvO planned and oversaw all laboratory analyses within the study. EB and TH provided through feedback on the study and manuscript. A-MH provided feedback on the written manuscript. J-WS and IvH were involved in patient management and provided valuable feedback on the manuscript. HtC oversaw the study setup and provided valuable feedback on the manuscript. HS oversaw the experimental setup and was involved in manuscript writing. BvB was involved in patient management and oversaw the study and manuscript writing. All authors contributed to the article and approved the submitted version.

## Funding

DCTC consortium is supported by funding from ZON-MW (Grant 10430012010004) and Dutch Thrombosis Foundation (Grant 2020_A).

## Conflict of interest

The authors declare that the research was conducted in the absence of any commercial or financial relationships that could be construed as a potential conflict of interest.

## Publisher's note

All claims expressed in this article are solely those of the authors and do not necessarily represent those of their affiliated organizations, or those of the publisher, the editors and the reviewers. Any product that may be evaluated in this article, or claim that may be made by its manufacturer, is not guaranteed or endorsed by the publisher.
